# Comparison between Linear and Branched Polyethylenimine and Reduced Graphene Oxide Coatings as a Capture Layer for Micro Resonant CO_2_ Gas Concentration Sensors

**DOI:** 10.3390/s20071824

**Published:** 2020-03-25

**Authors:** Alberto Prud’homme, Frederic Nabki

**Affiliations:** Department of Electrical Engineering, École de Technologie Supérieure, Montreal, QC H3C 1K3, Canada; Frederic.Nabki@etsmtl.ca

**Keywords:** polyethylenimine, micro-resonator, CO_2_ sensor, gas sensor, humidity sensor, reduced graphene oxide, coatings, mass sensor

## Abstract

The comparison between potential coatings for the measurement of CO_2_ concentration through the frequency shift in micro-resonators is presented. The polymers evaluated are linear polyethylenimine, branched polyethylenimine and reduced graphene oxide (rGO) by microwave reduction with polyethylenimine. The characterization of the coatings was made by using 6 MHz gold-plated quartz crystals, and a proof-of-concept sensor is shown with a diaphragm electrostatic microelectromechanical systems (MEMS) resonator. The methods of producing the solutions of the polymers deposited onto the quartz crystals are presented. A CO_2_ concentration range from 0.05% to 1% was dissolved in air and humidity level were controlled and evaluated. Linear polyethylenimine showed superior performance with a reaction time obtained for stabilization after the concentration increase of 345 s, while the time for recovery was of 126 s, with a maximum frequency deviation of 33.6 Hz for an in-air CO_2_ concentration of 0.1%.

## 1. Introduction

The measurement and capture of greenhouse gases such as CO_2_ have been a subject of research in recent decades due to their significant impact on the environment and to quality of life concerns. In combustion vehicles, industrial processes, biochemical processes etc., the measurement of CO_2_ concentration has become a critical factor in increasing process efficiency and reducing environmental impact [1,2]. Moreover, CO_2_ concentrations in human-occupied environments is an important metric that can significantly impact air quality.

The constant need to monitor the concentration of gases such as CO_2_ in open spaces or areas of common use among people, has encouraged the development of reduced cost, compact and low energy consumption measurement sensors. The market for gas sensors has grown significantly, having a market valued in more than USD 2 billion in 2018 with a predicted compound annual growth rate (CAGR) of 7.8% to reach in 2025. In this market, amongst the gas sensors with the greatest growth potential, are oxygen (O), carbon dioxide (CO_2_), and nitrogen oxide (NOX) sensors. [3]

It is currently possible to perform CO_2_ measurement with great precision and in a matter of seconds by means of commercial sensors with various measurement principles, which can be optical, resistive or capacitive, amongst others [4,5,6,7,8,9]. These sensors have the disadvantage of being bulky and relatively expensive. Consequently, alternative CO_2_ sensing solutions have been investigated in recent years [10,11,12].

The research on sensor miniaturization through microfabrication has shown that it is possible to obtain integrated measurement systems that are much smaller than commercial sensors today. In the case of CO_2_ sensors, optical sensors (e.g., infrared-based sensors) are prevalent due to their reduced response time, accuracy and durability. However, these sensors can occupy areas of up to 10 cm^2^, while a micromachined CO_2_ sensor with similar capabilities can occupy less than 10% of the area with a reduced cost [13].

Microelectromechanical systems (MEMS) resonators (or micro-resonators) used as mass sensors for measuring gas concentration have evolved significantly in recent years as a result of their sensitivity, low manufacturing cost and reduced footprint. This positions micro-resonator-based CO_2_ sensors as a potentially improved sensing technology [14,15]. However, such sensors have sensitivity to external factors such as temperature, humidity and pressure, which has limited the range of applications thus far [16,17].

To use micro-resonators as CO_2_ concentration sensors, it is necessary to add a surface coating, which has the function of capturing the gas molecules. This increases the mass of the micro-resonator, which in turn causes a reduction in the resonance frequency that can be monitored. This shift is proportional to the partition coefficient of the coating and the CO_2_ molecules [18,19]. 

The performance of the gas sensors depends on their selectivity, reaction and recovery times, and durability. These characteristics depend specifically on the capture coating, the gas selectivity being the most critical and complex of the parameters [20].

When addressing the capture of CO_2_, a material capable of capturing the greatest number of molecules is favored. These molecules are then trapped within the coating to prevent them from being released into the environment. One aims to find a coating that captures the CO_2_ molecules proportionally to the concentration in the environment, and that subsequently releases them when decreasing the environmental concentration, which is based on the coating’s coefficient of partition [21]. In this fashion, it is possible to increase and decrease the mass added to the resonator in a manner proportional to the environmental CO_2_ concentration, which shifts the resonance frequency within the operating range of the sensor [22]. Ideally, a linear frequency shift in response to the concentration variation is desirable. 

Multiple materials have been analyzed to be used for such coatings. Amongst these materials are the adsorbent coatings based on amines, which have been extensively studied, and which exhibit advantages such as their linearity in the adsorption of CO_2_, their regeneration under ambient temperatures and pressures, their high selectivity and their low sensitivity to humidity [23,24]. An example of this type of polymer is polyethylenimine (PEI). Lineal and branched PEI are polymers that have demonstrated a CO_2_ adsorption capacity of between 2 to 5 mmol/g. Their ability to adsorb and recover at atmospheric temperature and pressure has been demonstrated under laboratory conditions [23,25,26]. The selectivity of PEI as adsorbent of CO_2_ molecules has been extensively investigated, showing that their reaction with gases such as nitrogen is considered negligible, being an important advantage considering that air consists of more than 70% nitrogen [27,28,29]. The main research works on PEI as a CO_2_ adsorbent seek to characterize its maximum adsorption and recovery properties.

Reduced graphene oxide (rGO) has also been studied as a CO_2_ adsorbent, because of its large capture area allowing to store up to 8.10 mmol/g of CO_2_ at 273 K at low pressure [30]. Due to their properties, CO_2_ molecules tend to get trapped in the rGO and can be released through pressure reduction, temperature increase or chemical processes. The combination of rGO with polymers such as PEI can increase the volume of adsorbed gas and allow its release by reducing the concentration of CO_2_ in the environment. Such combinations could increase the efficiency of sensors based on the adsorption and recovery of gas molecules in relation to the partition coefficient.

Accordingly, this work aims to compare the performance of both branched and linear PEI sensing materials targeted for use in CO_2_ concentration sensors by micro-resonators operated in air. Additionally, the performance of PEI is evaluated in combination with rGO, obtained by microwave reduction of graphene oxide [31]. 

The paper is structured as follows: the preparation of the coatings as well as the deposition techniques on the quartz crystals are detailed in Section 2. Subsequently, the methodology and instrumentation used to carry out the characterization processes are defined in Section 2.1. This is followed by Section 2.2 in which the manufacturing process of the micro-resonator device and its operating parameters are presented. Section 3 presents the results of the characterization of the quartz crystals and the micro-resonator device, including the adsorption and recovery time and frequency shift at different levels of CO_2_ concentration, as well as the frequency shift in the presence of different levels of humidity. Section 4 discusses the results and compares them with other reported works. Finally, conclusions are presented.

## 2. Materials and Methods

The characterization of the CO_2_ adsorbing coatings aims to compare their adsorption capabilities, but also evaluate their suitability of being used as the surface coating that will be deposited onto a micro-resonator in order to implement a resonant CO_2_ sensor.

The CO_2_ concentration range analyzed was 0.05% to 1% dissolved in air, at a steady temperature that remained between 23 and 25 °C. Additionally, the coatings were characterized under different humidity levels ranging from 15 to 75 %RH with a constant CO_2_ concentration of 500 ppm.

For the initial characterization, quartz thickness monitor crystals of 6 MHz and 14 mm in diameter were used due to their high Q-factor of more than 15,000 at atmospheric pressure [32] and their stability to temperature variations. Branched and linear PEI coatings were analysed, and the one that yielded the best results was used to integrate with rGO to characterize its impact on adsorption performance.

Once the best performing coating was identified, a transducer proof-of-concept was implemented. This was done by using an electrostatic micro-resonator coated with the adsorbing material. The coated micro-resonator was exposed to varying CO_2_ concentrations and the resulting resonant frequency shifts of the structure were monitored.

### 2.1. Coating Preparation

**Figure 1:**
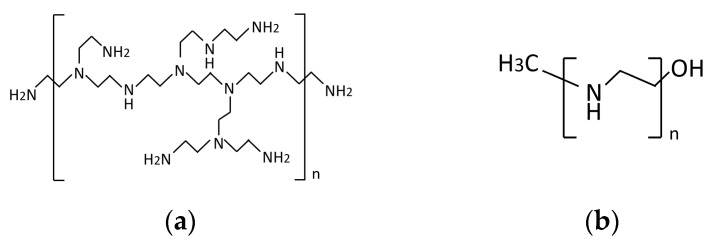
(**a**) Branched polyethylenimine molecule [33], and (**b**) lineal polyethylenimine molecule [34].

Branched PEI and linear PEI are shown in Figure 1. The branched PEI has an average Mw of ~25,000 by LS, an average Mn of ~10,000, and it was acquired through Sigma-Aldrich (San Luis, Misuri, Estados Unidos). The linear PEI has an average Mn of ~10,000, PDI ≤ 1.3, and was also acquired through Sigma-Aldrich.

**Figure 2:**
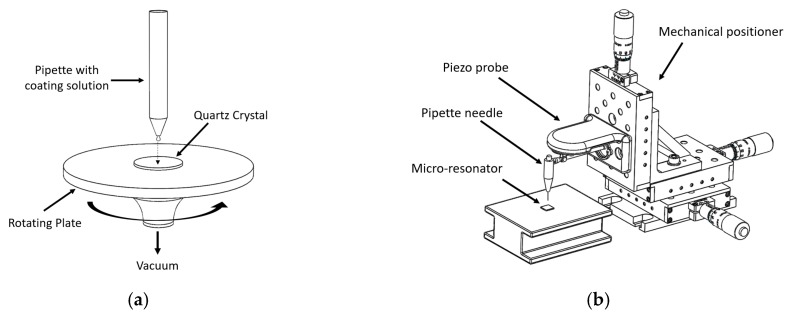
Coating deposition technique, (**a**) spin coater for the quartz crystal used for initial coating tests, (**b**) micro-drop deposition by piezo probe used for the micro-resonator sensor.

Due to the high viscosity of the branched PEI and the granulated state of the linear PEI, a specific preparation process was necessary to obtain solutions suited to the deposition of a thin film on the quartz crystals. Similar processes used for the preparation of the solutions have been previously published in other works [29,35]. Figure 2 shows how the solutions were deposited onto the quartz crystals, characterized in Section 3.1, Section 3.2 and Section 3.3 or onto the micro-resonator, characterized in Section 3.4. A spin coating system was used for the former while a custom-designed piezoelectric micro positioning stage with a pipette was used for the latter. 

The solution of branched PEI was prepared in these steps:

1. Dissolution of 10 ml of branched PEI in 10 ml of distilled water at 40 °C.
2. Sonification of the mixture for 2 hours at 40 °C.
3. Maintain the solution in a water bath at >40 °C (before deposition onto the quart crystal).

The crystal used for the characterization was a gold-plated quartz of 14 mm diameter and a center frequency of 5.950 MHz from Inficon. The deposition onto the crystal was done using a spin coater as shown in Figure 2a using the following sequence:

1. 500 rpm for 5 seconds while a drop of branched PEI solution is deposited.
2. 2500 rpm for 15 seconds to remove the solution in excess.
3. 5000 rpm for 30 s.
4. The deposited crystal with the branched PEI was maintained for 24 hours in controlled ambient conditions and a maximum of 500 ppm of CO_2_.

In the case of the Lineal PEI, the dissolution requires a minimum temperature of 50 °C, so the solution was prepared at 55 °C, and during the entirety of the process, the temperature was maintained at that level. The procedure is listed below:

1. Dissolution of 0.25 gr of Lineal PEI in 10 ml of water at 55 °C.
2. Sonification of the mixture for 1 hour at 55 °C, verifying the level of water periodically.
3. Leave the solution at room temperature (25 °C ± 1 °C) for 24 h, at this temperature the solution will turn into a white hard paste.

The deposition was done using the same spin coater, but the crystal should be at a temperature of at least 40 °C in order to reduce the solidification of the solution when in contact with the crystal. This ensures that the deposited layer is regular and suited to the application. Accordingly, the deposition of the linear PEI was done following these steps:

1. Heat the crystal with a Peltier cell with the hot side in contact with the crystal to increase the temperature to ~50 °C, and then remove the Peltier cell and turn on the spin coater as rapidly as possible.
2. 1800 rpm for 10 seconds while a drop of linear PEI solution at 55 °C is deposited.
3. 2500 rpm for 45 s.
4. The deposited crystal with the linear PEI was maintained for 24 hours in controlled ambient conditions at a maximum of 500 ppm of CO_2_.

Once the crystals with both coatings were characterized, the coating with the best performance was selected considering the characteristics mentioned above. Subsequently, a third coating was produced using rGO, which was obtained by microwave reduction. This sought to increase the available area of adsorption and achieve a greater capture of CO_2_ molecules, resulting in increased sensitivity [36,37]. As will be detailed in Section 3, characterization data indicates that the linear PEI performs better than the branched PEI for CO_2_ sensing.

**Figure 3:**
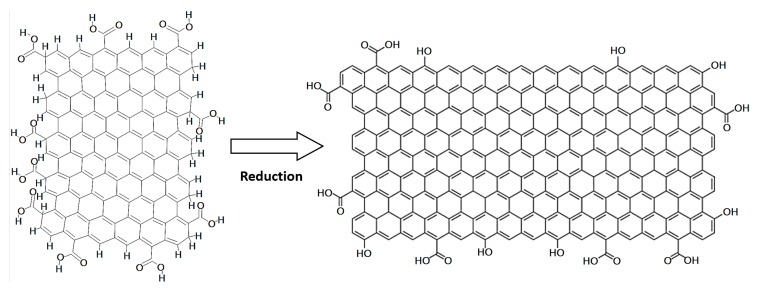
Reduced graphene oxide by microwave reduction.

Figure 3 shows the transformation of the graphene oxide (GO) treated by microwave radiation generating a considerable amount of heat followed by the reduction and then exfoliation of the GO generating the rGO.

The third coating that includes linear PEI and rGO was produced using methods that influenced this work [38,39]. The process is described below:

1. Sonification of 0.5 ml of linear PEI solution with 20 ml of distilled water for 10 minutes at 50 °C verifying the level of water periodically.
2. Sonification of 0.5 g of graphene oxide within the solution for 1 hour at 50 °C verifying the level of water periodically.
3. Filter the solution with graphene.
4. 3 microwave reduction cycles of 7 seconds each at a 600 W power with 20 seconds between each cycle.
5. Sonification of the reduced graphene oxide with the original linear PEI solution in a volumetric proportion of 1:1 for 3 hours at 55 °C verifying the level of water periodically.
6. Leave the solution at room temperature (25 °C ± 1 °C) for 24 h, at this temperature the solution will turn into a dark gray paste.

**Figure 4:**
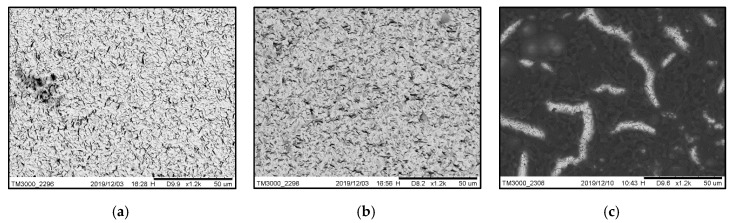
SEM image of the quartz crystal coated with the (**a**) branched PEI layer, (**b**) linear PEI layer and (**c**) rGO with linear PEI layer.

For the deposition of this solution, the same process used for the deposition of the linear PEI coating was followed, however, it was important to verify that there were no agglutinated residues of rGO on the surface of the quartz. Figure 4 shows the SEM micrographs of the quartz crystal’s surface for the three coatings investigated. This method is widely used to allow for the observation of the quality of the deposited material, and for the verification of the area of the quartz crystal that has been coated [40,41]. The uniformity is similar between the deposited PEI coatings, which is a critical factor to be able to make an equitable comparison and to maintain the high Q factor of the crystals. In the case of the coating of rGO + PEI, the deposition of the rGO is evident and the uniformity it is equivalent to the other coatings.

**Figure 5:**
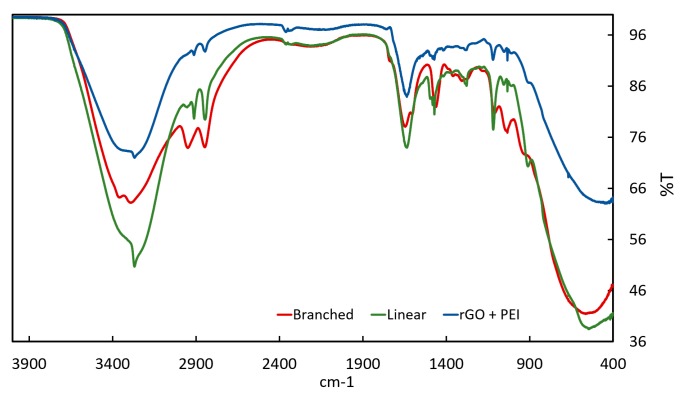
Infrared (IR) absorption spectroscopy result for the three deposited coatings on the quartz crystals.

In Figure 5, infrared (IR) absorption spectroscopy of the coating materials was carried-out in order to verify the integrity of the materials. For the coating formed by rGO + PEI, the presence of the linear PEI with the influence of the rGO is observed. The coatings of linear and branched PEI exhibited a typical response for these kind of polymers [42]. Notably, the characteristic peaks of the PEI that can be seen at 2799 cm^−1^ are caused by mode CH_2_-SS, at 3279 cm^−1^ caused by mode NH, and at 1644 cm^−1^ and 1599 cm^−1^ caused by C=N and N–H [42,43,44]. For the reduced graphene oxide, peaks are observed at 1730 cm^−1^ caused by C=O, at 1414 cm^−1^ caused by the carboxy C–O and at 1622 cm^−1^ caused by aromatic CC [38].

### 2.2. Test Setup

**Figure 6:**
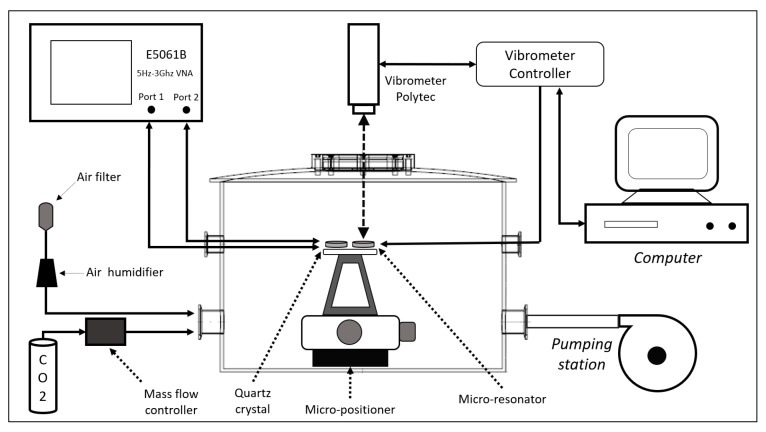
Diagram of the characterization setup.

For the control of the pressure, temperature, humidity and CO_2_ concentration conditions, an environmental test chamber was used, as shown in Figure 6. The S-parameter S21 measurements performed for the characterization of the crystals with the coatings were done using a Vector Network Analyzer (VNA) Keysight-E5061B (Santa Rosa, CA, USA). This was done to determine their resonant behavior in the presence of CO_2_ and humidity. In the case of the electrostatic micro-resonator, because the low Q-factor and the electrostatic actuation, a VNA could not be used. As such, the measurements were made using a Polytec OFV-534 (Waldbronn, Germany) vibrometer in closed loop with an OFV-2570 controller (Waldbronn, Germany). 

The CO_2_ introduction and concentration level control were made with a constant low flow of air to maintain the atmospheric pressure and obtain a smooth increasing and decreasing CO_2_ concentration in the chamber through the pumping station. The air introduced to the chamber comes from the laboratory where the temperature and humidity are automatically controlled, in a range of ±2.5 %RH and ±0.5 °C. Even so, during the tests the humidity, temperature and CO_2_ levels in the laboratory were constantly supervised to avoid unexpected changes that could affect the tests. Due to the susceptibility of the coatings to these parameters, any variation would be detected immediately so the test could be eliminated and repeated.

The full setup was designed to be able to develop a complete characterization in almost a fully automated process. The VNA and vibrometer were integrated with a MATLAB (Natick, Massachusetts, USA) platform that registered and controlled the process, while the micro-positioner was used for the alignment of the MEMS resonator with the vibrometer laser.

**Figure 7:**
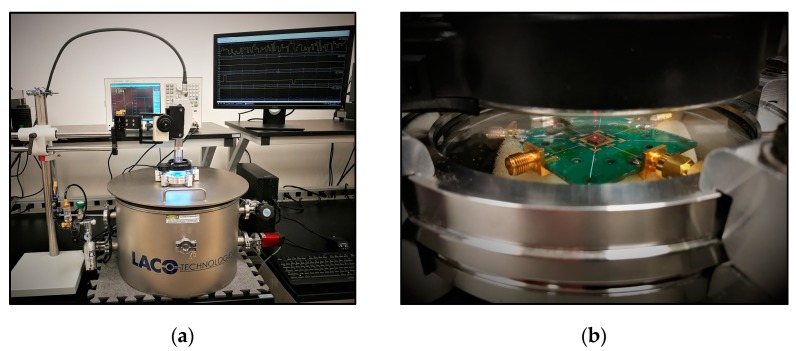
(**a**) Setup for the quartz crystals and the resonator characterization, (**b**) close-up of the micro-resonator during the characerizacion with the laser from the vibrometer visible on the micro-resonator die.

The characterization equipment is shown in Figure 7. The fully integrated setup allows the characterization of various types of sensors. In Figure 7b, a close-up of the vibrometer with the micro-resonator seed through the viewport of the chamber is shown. This allows to characterize the low Q-factor micro-resonators at different pressures, temperatures and humidity, while electrostatically actuating them.

### 2.3. Micro-resonator Characteristics

**Figure 8:**
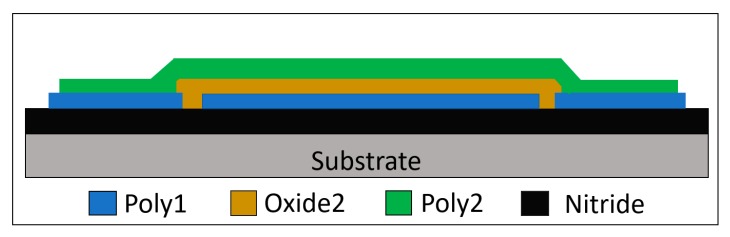
Cross-section of the micro-resonator with oxide as sacrificial layer.

The micro-resonator that was used was a diaphragm structure to increase its mechanical stability and provide enough deposition area for the coating, although the Q-factor was reduced as a result of this geometry. The resonator device, with cross-section shown in Figure 8, was manufactured using the MEMSCAP PolyMUMPS (Crolles Cedex, France) commercial fabrication process. A polysilicon layer (Poly2) was used as the structural layer of the diaphragm and it was anchored at its sides. An underlying polysilicon layer (Poly1) allowed for the electrostatic driving of the resonator. An oxide layer (Oxide2) between both polysilicon layers was used as a sacrificial layer which was chemically removed to release the structure. The achieved gap between the diaphragm and the underlying driving electrode was of 0.75 µm. Such a small gap allowed for a reduced driving voltage requirement. The CO_2_ absorbing coating was deposited above the diaphragm post-fabrication by processing the dies received from the foundry. This was done using a micro-needle that was carrying at its tip a small drop of the coating as shown in Figure 2b. With micro-positioners the needle was placed at the center of the micro-resonator, slowly descending until the polymer touched the surface of the resonator. After removing the needle, the deposited size of the coating drop is estimated to be of around 40 µm in diameter.

**Figure 9:**
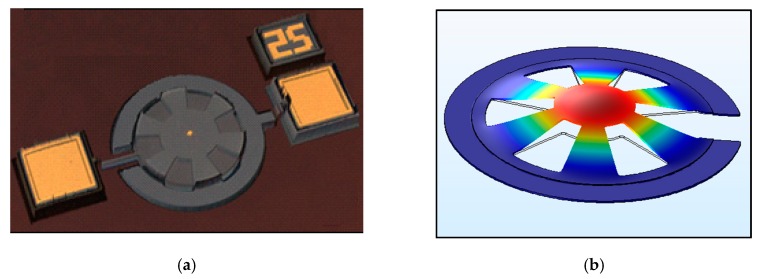
(**a**) Three-dimensional (3D) view of the real micro-resonator obtained by the Olympus (Shinjuku, Tokyo, Japón) LEXT OLS4000-Confocal microscope, (**b**) Eigenfrequency FEM COMSOL simulation of the first mode of resonance of the microelectromechanical systems (MEMS) resonator.

Figure 9 shows the 3D view of the imaged micro-resonator and its mode-shape simulation carried out using COMSOL (Stockholm, Sweden). The resonator is a diaphragm with external opening to reduce the damping caused by the air flow between the plates and the increase of the amplitude of resonance. The diameter of the resonator is 200 µm and it is 1.5 µm thick. 

The Eigenfrequency finite element method (FEM) mode shape simulation of the resonator estimated a resonance frequency of the first mode at 1.16 MHz. The measured resonant frequency of the fabricated resonator obtained by using the vibrometer was of 1.15 MHz, matching well with the simulations. The measured Q-factor was of 8 at atmospheric pressure and of 300 at a 1 mTorr ambient pressure. Once the micro-resonator was characterized, the Linear PEI coating was deposited following the process previously mentioned. The measured resonant frequency of the micro-resonator with the coating was of 1.139 MHz. The decrease of resonant frequency is due to the mass loading of the coating on the resonant structure.

## 3. Results

The characterization of the coatings deposited in the quartz crystals focused on three main factors, the maximum frequency shift before stabilizing in the presence of a higher concentration of CO_2_ in a range of 0.05 to 1%, the stabilization time and the recovery time needed to return to the starting point. If during the recovery the resonant frequency did not reach the initial value, the maximum time was considered to be that required to stabilize the recovery response. In that case, the final frequency value achieved was considered as hysteresis. The impact of air humidity in a range of 15% to 75% RH at a CO_2_ concentration of 500 ppm was also analyzed.

The measurements were made at three different CO_2_ concentrations of 0.1%, 0.5% and 1% to analyze the linearity of the frequency deviation. Comparisons were made between the different coatings at the same concentrations in order to compare the maximum deviation frequency, adsorption time and recovery time.

During this test the CO_2_ was introduced until the desired concentration was reached in the chamber, during this process the coatings began to adsorb the CO_2_ molecules, and this was reflected in the deviation of the oscillation frequency of the quartz crystal. Throughout the test, the pumping equipment maintained a constant flow of air output, so that the CO_2_ had to be continuously introduced to maintain the concentration. This allowed the CO_2_ concentration to be decreased naturally by closing the CO_2_ valve without any alteration in the pressure or abrupt changes in the atmosphere inside the chamber.

### 3.1. Linear and Branched Polyethylenimine Coatings Characterization

The first characterization was made with the coatings formed by linear and branched PEI, to define which one exhibited better performance, and to later use it to make the solution with the rGO.

Once the frequency deviation stabilized, the introduction of gas was stopped in order to decrease the concentration of CO_2_ inside the chamber to the external value of 500 ppm. Once the quartz crystal returned to its initial frequency, the CO_2_ was introduced again to the next concentration value.

The test results are shown below. Within each graph, the CO_2_ concentration level inside the test chamber is indicated in colored lines, the 0.1% in red, 0.5% in blue and 1.0% in green. The frequency deviation of the crystal is shown with the black line. As can be seen, the CO_2_ concentration level in the chamber is increased rapidly until reaching the level determined for the test. Once the adsorption phase by the coating is finished, the CO_2_ level is rapidly decreased to analyze the recovery time of the coating that is in characterization. Each coating is analyzed individually to facilitate the interpretation of the information provided by each test, then a comparison of the three coatings is made in detail.

**Figure 10:**
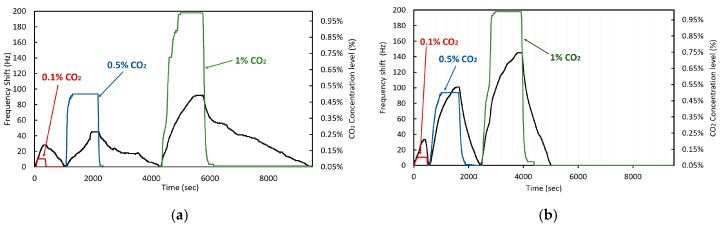
Quartz crystal frequency shift in response to different CO_2_ concentration cycles for (**a**) branched PEI coated crystal, and (**b**) linear PEI coated crystal.

In Figure 10, the reaction of both coatings complies with the adsorption theory and a deviation of the frequency is seen in response to the introduction of CO_2_. However, the frequency shift amount and recovery time is different for each material. In all cases, the frequency shift obtained using the linear PEI coated crystal generated between 15% and 30% greater deviation than the branched PEI coating. An identical time scale has been used in both plots to compare the adsorption time between the coatings. The recovery time was between 2 to 3 times faster for the crystal coated by linear PEI.

**Figure 11:**
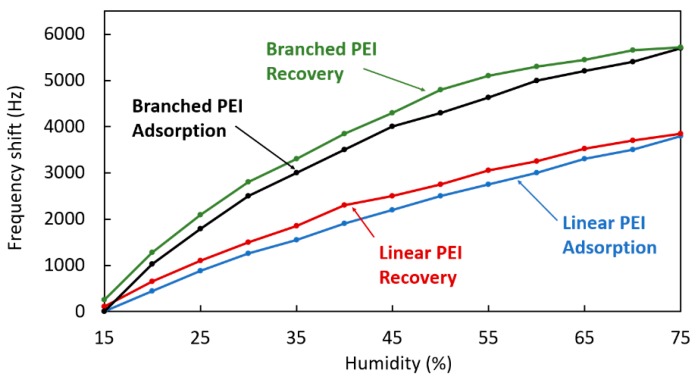
Frequency shift of branched PEI and linear PEI coated crystals at different humidity levels while in absorption and recovery.

In Figure 11, the frequency shift in response to humidity changes is plotted for both the branched and linear PEI coated crystals. The CO_2_ concentration in these cases is constant at 500 ppm. In both cases, the frequency deviation can be considered as linear in the range shown, however, the deviation is much greater in the case of the branched PEI coated crystal, showing more than a 60% increased shift compared to the linear PEI coated crystal. The measurement was carried out for both increasing (adsorption) and decreasing (recovery) humidity levels and both coatings exhibited similar hysteresis.

The results above indicate that the linear PEI coating has better performance in comparison to the branched PEI. This is the case in terms of the frequency shift amount (sensitivity), reaction time and susceptibility to ambient humidity. Therefore, the linear PEI solution was chosen to be integrated with the reduced graphene oxide as the third coating for investigation.

### 3.2. Linear Polyethylenimine with Reduced Graphene Oxide Coating Characterization

**Figure 12:**
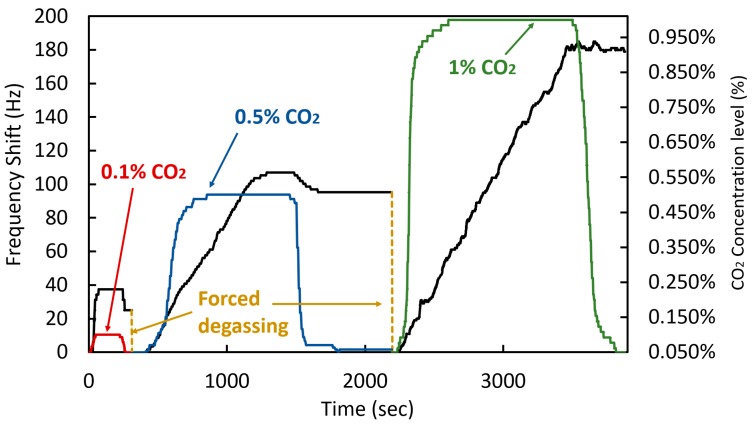
Frequency shift of the quartz crystal with linear PEI with rGO in response to different CO_2_ concentration.

Through the methodology discussed in Section 2, the linear PEI with rGO was deposited onto a quartz crystal to characterize its performance. Figure 12 shows the characterization results of the coated crystal. As expected, the coating formed by linear PEI and rGO showed the same adsorption tendency, however, during the recovery process, the coating does not allow the adsorbed molecules to be released readily. Indeed, the desorption was limited to about 15% when dropping the CO_2_ concentration, and very little recovery was observed at the higher 1% CO_2_ concentration. This phenomenon occurs due to the combination of the rGO with the polymer-based amines, which has shown excellent properties of capturing CO_2_. Accordingly, external processes are required for recovery, either by increasing temperature and/or decreasing the atmospheric pressure in order to force degassing [45,46]. Accordingly, forced degassing was carried-out here in order to reset the coating between CO_2_ concentration cycles. It was necessary to place the coated quartz crystal at a 10 mTorr vacuum for 30 minutes and subsequently for 2 hours in ambient conditions at no more than 500 ppm CO_2_ concentration in order to reset the coating. The linear PEI with rOG coated crystal exhibits a frequency deviation in response to humidity variations that is similar to that of the quartz crystal coated with branched PEI.

Note that the CO_2_ capture behavior of the PEI with rOG may be suitable for sensors that aim to detect whether a CO_2_ concentration occurs in an environment over time and retain this information. Such a sensor may be read periodically in order to determine if a high enough CO_2_ concentration was reached or not at some point in time. Because this type of application is not part of objective of this work, no quantitative study retention time or loss level tests were carried out for prolonged periods and these should be further investigated to confirm the applicability of PEI with rOG in such applications. However, by considering others published works on coatings based on amines with graphene or carbon nanotubes, and where it is concluded that it is necessary to expose the coating to a vacuum and/or a high temperature process for recovery, it is possible that the coating in this work would yield long-term CO_2_ retention and be suitable to the aforementioned application [47,48].

### 3.3. Performance Comparison between the Coatings

**Figure 13:**
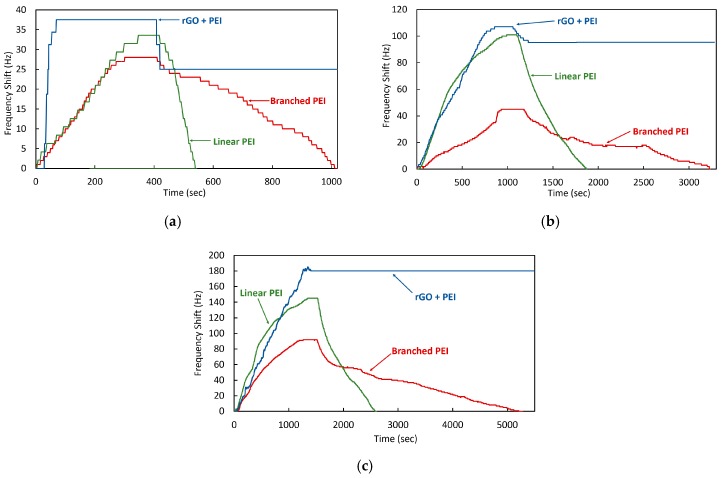
Response of the three different coatings at CO_2_ concentrations of (**a**) 0.1%, (**b**) 0.5%, and (**c**) 1%.

The performance comparison of all coated quartz crystals under the same CO_2_ concentration was carried out in order to analyze the total frequency deviation and reaction times of each. The results at different concentrations are shown in Figure 13.

The performance of the coatings when compared by levels of CO_2_ concentration show the same tendency to adsorb the gas molecules, however, the frequency shift rate showed a disproportional increase between branched PEI, linear PEI and linear PEI + rGO due to CO_2_ concentration. This is outlined in Table 1. 

The difference in the frequency deviation of each coating can be observed, which shows that Linear PEI with + rGO has the greatest adsorption capacity, an advantage of the inclusion of rGO. This frequency shift is followed by that of the linear PEI. Table 2 shows the linearized value of the frequency deviation per unit of ppm of CO_2_, outlining the sensitivity of each coating. The PEI+rGO exhibits a 25% greater adsorption capacity than the linear PEI coating, which is 45% more adsorbent than the branched PEI coating.

**Figure 14:**
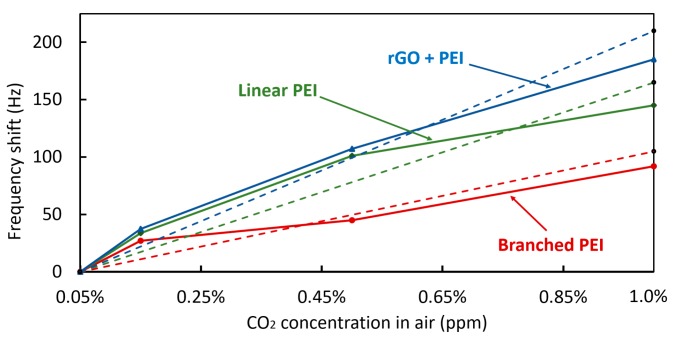
Frequency shift of each coating from 0.05% to 1% of CO_2_.

The frequency shift in response to the CO_2_ concentration is shown in Figure 14. The ideal response for this type of sensor would be linear for any level of CO_2_ concentration, however, the PEI has a saturation limit of up to 2 to 3 mmol/g for the branched and linear PEI. Accordingly, concentrations ranging from 0 to 0.2% yield a linear response. However, at higher concentration values, the proportion of adsorption capacity is progressively reduced until it reaches its saturation point [49,50]. In the case of the rGO with linear PEI, a higher adsorption capacity of 8.10 mmol/g of CO_2_ at 273 K and low pressure has been reported [30,40]. Due to this, the results obtained show a higher adsorption ratio from 0.05% to 0.15%, followed by a progressive reduction in the frequency deviation.

**Figure 15:**
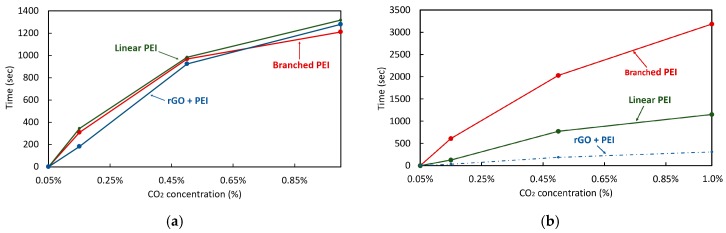
(**a**) Adsorption time for each coating at different CO_2_ concentrations, (**b**) recovery time for each coating at different CO_2_ level. The recovery time for the linear PEI with rGO reflects only the time to stabilize the desorption to the captured CO_2_ value.

The adsorption time for each coating was also characterized and is plotted in Figure 15a. The adsorption was defined as the time for the frequency shift to go from 0% to 90% of its steady state value. The absorption time of the coatings maintains a linear increase up to 0.5% of CO_2_ concentration, subsequently the adsorption time it is reduced but still maintaining the same proportion among all the coatings evaluated. The adsorption times for each coating are summarized in Table 3.

The branched and linear PEI exhibit similar adsorption times over the range of concentrations measured. In the case of the rGO + PEI, the adsorption time for low concentrations is shorter than that observed with the other coatings because of the high absorbance capacity of the rGO [30]. For all coatings, after the CO_2_ concentration is sufficiently increased, the slope of the adsorption time begins to decrease, this is attributed to the reduction of the adsorption capacity of the coating at higher concentrations [51,52].

The recovery times of each coating was also characterized and is plotted in Figure 15b and summarized in Table 4. The recovery time for the PEI with rGO coating was not characterized in the same fashion because of the coating’s capture of CO_2_ precluding from a return of the resonant frequency to its initial condition without forced degassing. The recovery time of the coating formed by branched PEI is more than 3 to 5 times longer than that of linear PEI, which is an important characteristic to consider for sensing applications.

**Figure 16:**
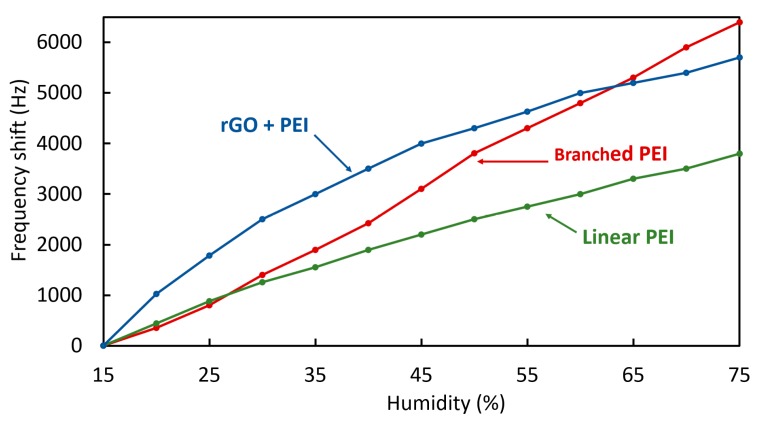
Frequency shift of the coatings at different range of air humidity from 15 to 75 %RH.

Figure 16 shows the frequency shift of the quartz crystals in response to different levels of humidity. The behavior of the branched PEI and lineal PEI is linear, while the PEI with rGO shows a reduced adsorption as the humidity level increases. Overall linear PEI shows lower sensitivity to humidity variations. 

Once the results obtained from the tests of characterization of the coatings are analyzed, it can be concluded that the formed by linear PEI presents qualities and superior performance for the type of application for which it has been proposed. Therefore this will be the coating to be used in the MEMS resonator to perform the proof of concept.

### 3.4. Micro-resonator CO_2_ Sensor Proof-of-concept

**Figure 17:**
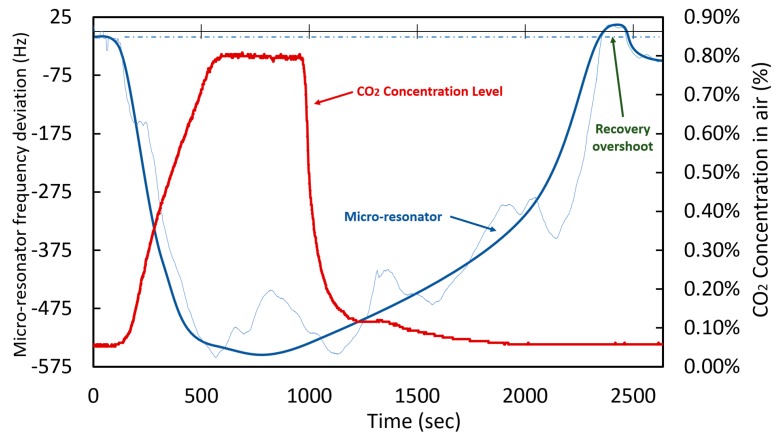
Response of the coated micro-resonator to a 0.8% CO_2_ concentration variation.

Following the above methodology, the electrostatic diaphragm micro-resonator described in Section 2.3 was coated with the linear PEI solution, which exhibited the overall better behavior for a proof-of-concept. The micro-resonator was first characterized to determine its behavior before and after a CO_2_ concentration variation. The adsorption and recovery frequency shift response of the micro-resonator to a 0.8% CO_2_ concentration increase is shown in Figure 17. The nominal resonant frequency of the micro-resonator is of 1.139 MHz.

The coated micro-resonator shows a behavior that is similar to that obtained by the quartz crystal. However, the low Q-factor decreases the accuracy with which measurements can be made. Even so, a deviation shift of 0.0675 Hz/ppm was attained which compares favourably to the quartz crystal-based sensors. Another important factor to mention is the overshoot that appears in the last phase of the recovery, where the value of the frequency exceeds the original value by approximately 35 Hz before stabilising a few minutes later. 

The observed overshoot is attributed to the kinetics of the gas molecules adsorption in linear PEI because generally the CO_2_ is adsorbed on the surface of the material and then diffuses into the bulk of the absorbent. This causes the stabilization process to not always be progressive resulting in overshoots [51,53]. Moreover, in high concentrations (>20%) and for long periods, the capacity of the adsorbent to release the molecules is reduced and hysteresis is generated.

**Figure 18:**
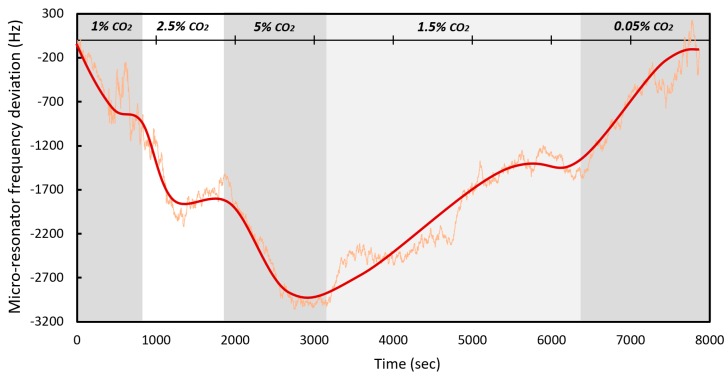
Behavior of the micro-resonator over time at different concentrations of CO_2_.

The micro-resonator was also characterized by performing a cycling of different CO_2_ concentrations in order to determine its stability throughout each concentration period. For this, CO_2_ was introduced to obtain a 1% concentration for a period of 15 minutes, followed by 2.5% for an equal period of 15 minutes, and 5% for a period of 20 minutes. Subsequently the concentration was reduced to 1.5% for a period of 50 minutes and finally 0.05% for 30 minutes. Note that the initial CO_2_ concentration before the test was of 0.05%. The resulting response is shown in the Figure 18.

During this test the resonator was able to maintain the upward and downward trend adequately, both in reaction time and in frequency deviation. Again, the overshoot can be seen at each stabilization point. On this occasion, the final hysteresis was approximately 55 Hz. Table 5 lists the frequency shift and ppm/Hz variation achieved over each phase. An absolute value ppm/Hz average of all the cycles was also calculated to be of 0.0646 Hz/ppm. This is consistent with the 0.0675 Hz/ppm value observed in the prior single concentration test reported above. 

## 4. Discussion

The results obtained have shown the possibility of using PEI as a coating layer for CO_2_ sensors. Linear PEI shows superior performance mainly during the recovery phase over [49]. Table 6 compares the performance of the coatings evaluated in this work to other previously published works that consider adsorbing coatings for CO_2_ concentration sensors as well. 

The performance obtained by the lineal PEI is similar than that reported using acrylonitrile–styrene copolymer (AS3), where the recovery time for the lineal PEI coating is slower by 1.85 minutes. The results obtained from the coating formed by linear PEI with rGO showed results not initially anticipated, however, it opens the opportunity to develop in depth the study of the capabilities of such a coating as a CO_2_ gas sensor for industrial and/or environmental applications. It can also be studied as an adsorbent coating for sensors where it is desired to retain the highest concentration value recorded during a given time period. The linear PEI with rGO recovery process must be further studied, as it would be possible to add an integrated heater to the micro-resonator structure in order to reset the sensor. This could allow to benefit from the higher adsorption capabilities of that coating while mitigating its CO_2_ capture limitation. Interestingly, the increased sensor response of the diaphragm micro-resonator sensor proof-of-concept presented in comparison to the linear PEI coated quartz (i.e., −474 ppm vs. −16.8 ppm) illustrates the advantage of miniaturizing the resonant structure of the sensor.

During the development of this work, the ambient temperature was maintained in a controlled manner at 25 ± 1 °C and the ambient humidity was maintained between 20 and 35 %RH. It can be relevant to perform a similar study with more tightly controlled conditions as ambient conditions may influence the coatings performance. 

Ultimately, the sensitivity to humidity of all of the coatings will require the use of calibration alongside a humidity sensing device that is not sensitive to CO_2_ in order to implement a reliable sensing device.

The micro-resonator used in this study could demonstrate the sensing operation and the miniaturization capabilities of such a device, however, as the Q-factor was relatively too low, the device makes it difficult to obtain high-precision measurements without significant averaging. Accordingly, a higher-Q micro-resonator structure should be considered.

As previously commented, the measurement of CO_2_ concentration has multiple applications with different requirements in sensing range and reaction time. Among some applications such as the monitoring of exhaust gases from a combustion engine, the reaction time must be as short as possible. In this case, IR sensors are used with reaction time can be as low as between 5 to 30 ms, while a resistive sensor for the same application can take up to 30 seconds, in both cases with sensors capable of measure until 90% of CO_2_ [60,61]. There also exists applications that do not require such short reaction times such as CO_2_ monitoring in the environment, which can range from a few seconds to minutes, with measuring ranges of less than 10% [62]. Accordingly, the results obtained in this work determine that the use of PEI-based coatings for the measurement of the CO_2_ concentration is limited to applications such as atmospheric sensing, whether for indoor or open areas. This is due to the reaction time, mainly in the recovery stage. The detection of the sharp increase in CO_2_ in an accelerated form such as a fire or laboratory safety sensor can be another application where this coating is used.

## 5. Conclusions

The purpose of this work was to study different CO_2_ adsorbing coatings and to assess whether a micro-resonator CO_2_ gas sensor could be implemented. Linear or branched PEI were considered as the adsorbent coating. Additionally, a linear PEI with rOG coating was also studied.

Results showed congruence with previously published studies and has opened the possibility of deepening its application in micro-resonators. Linear polyethylenimine coating has stood out for its capture and recovery properties, as well as its reduced hysteresis and reduced sensitivity to humidity. Linear PEI with rOG has shown interesting adsorption capabilities but captures the CO_2_ molecule which requires an active reset mechanism, making this coating ill-suited for some application. However, the coating can be further investigated to integrate a reset mechanism within the sensor.

This work also presented a proof-of-concept micro-resonator based CO_2_ gas sensor operating with a linear PEI coating. The sensor represents a highly integrated solution which could be batch fabricated, as the underlying resonant structure is fabricated using a commercial MEMS fabrication process. This work thus represents an initial effort towards achieving a highly integrated low-cost CO_2_ sensor structure and future work could focus on integrating electronics to the structure in order to obtain a full-featured sensor device. 

## Figures and Tables

**Table 1:** Frequency shift (in Hz) of each coating at different concentration of CO_2_.

	Branched PEI	Linear PEI	Linear PEI + rGO
0.1% CO_2_	27	33.6	37.5
0.5% CO_2_	45	101	107
1% CO_2_	92	145	185

**Table 2:** Linearized coefficient of the frequency shift by ppm of CO_2._

Branched PEI	Linear PEI	Linear PEI + rGO
0.011 Hz/ppm	0.016 Hz/ppm	0.020 Hz/ppm

**Table 3:** Adsorption time for each coating.

	Branched PEI	Linear PEI	rGO + PEI
**0.1% CO** **_2_**	309 sec	345 sec	150 sec
**0.5% CO** **_2_**	969 sec	984 sec	924 sec
**1.0% CO** **_2_**	1210 sec	1317 sec	1281 sec

**Table 4:** Recovery time for each coating.

	Branched PEI	Linear PEI	Linear PEI with rGO
0.1% CO_2_	609 sec	126 sec	n/a
0.5% CO_2_	2031 sec	771 sec	n/a
1.0% CO_2_	3187 sec	1152 sec	n/a

**Table 5:** Frequency shift of the micro-resonator at different CO_2_ concentrations.

Concentration Period	Frequency Shift (Hz) from the Last Level	Hz/ppm
**1%**	750	0.075
**2.5%**	1050	0.07
**5%**	1200	0.048
**1.5%**	−1600	−0.045
**0.05%**	−1350	−0.085
	*Average (Hz/ppm)*	0.0646

**Table 6:** Comparison of the studied CO_2_ adsorbing coatings with other published works.

	Sensing Material	CO_2_ Level	Sensor Output	Sensor Response (ppm)	Test Device	Adsorption Time (min)	Recovery Time (min)
**This work**	Linear PEI	0.5%	Frequency	−16.8	6 MHz gold quartz crystal	16.4	12.85
	Branched PEI	0.5%	Frequency	−7.5	6 MHz gold quartz crystal	16.15	33.85
	rGO + Linear PEI	0.5%	Frequency	−17.8	6 MHz gold quartz crystal	15.4	n/a
	Linear PEI	0.8%	Frequency	−474	1.13 MHz diaphragmmicro-resonator	13.5	20.4
**Sun et al.** [29]	PEI **	0.5%	Frequency	−3.33	7 MHz AT-cut quartz crystal	11.7	20.7
**Sun et al.** [29]	PEI ** + Starch	0.5%	Frequency	−11.66	7 MHz AT-cut quartz crystal	18.9	21.6
**Muraoka et al.** [54]	acrylonitrile–styrene copolymer (AS2)	20%	Frequency	−7.5	10 MHz AT-cut quartz crystal	6	11
**Muraoka et al.** [54]	acrylonitrile–styrene copolymer (AS3)	20%	Frequency	−20	10 MHz AT-cut quartz crystal	7	11
**Gomes et al.** [55]	1,2-diaminoethane	5 ml	Frequency	−25	9 MHz AT-cut quartz crystal	N/A	N/A
**Doan et al.** [50]	Linear PEI with polyelectrolytes	1.0%	Impedance	3e6	Coated silicon chip	16.5	41.6
**Ing et al.** [56]	Carbon nanotubes (MWNTs)	20%	Permittivity	−1800	Coated plates	1.1	1.3
**Ando et al.** [57]	Graphene with PEDOT/PSS	0.2%	Impedance	2050	Coated plates	2	N/A
**Boudaden et al.** [58]	Branched PEI with Silica	0.1%	Capacitance	−105e3	Coated plates	5	8
**Ma et al.** [59]	PEI with Vinyl Alcohol	15.4%	Wavelength	4000	Optic fiber coated	0.5	0.2

** The PEI type was not indicated, however considering the properties and the solution preparation process it is assumed that branched PEI was used.

## References

[1] Espinal L., Poster D.L., Wong-Ng W., Allen A.J., Green M.L. (2013). Measurement, Standards, and Data Needs for CO2 Capture Materials: A Critical Review. Environ. Sci. Technol..

[2] Mahyuddin N., Awbi H. (2012). A Review of CO2 Measurement Procedures in Ventilation Research. Int. J. Vent..

[3] Gas Sensor Market Size, Share & Trends Analysis Report By Product (CO2, NOx, CO, O2 Sensors), by Technology (Semiconductor, Infrared), by End Use (Building Automation & Domestic Appliance, Industrial), and Segment Forecasts, 2019–2025. https://www.grandviewresearch.com/industry-analysis/gas-sensors-market.

[4] Fernández-Ramos M.D., Moreno-Puche F., Escobedo P., García-López P.A., Capitán-Vallvey L.F., Martínez-Olmos A. (2020). Optical portable instrument for the determination of CO2 in indoor environments. Talanta.

[5] Ghosh A., Zhang C., Shi S., Zhang H. (2019). High temperature CO2 sensing and its cross-sensitivity towards H2 and CO gas using calcium doped ZnO thin film coated langasite SAW sensor. Sensors Actuators B Chem..

[6] Hsu K.C., Fang T.H., Hsiao Y.J., Chan C.A. (2020). Highly response CO2 gas sensor based on Au-La2O3 doped SnO2 nanofibers. Mater. Lett..

[7] Lin Y., Fan Z. (2020). Compositing strategies to enhance the performance of chemiresistive CO2 gas sensors. Mater. Sci. Semicond Process.

[8] Serban B., Kumar A.S., Costea S., Mihaila M., Buiu O., Brezeanu M., Varachiu N., Cobianu C. (2009). Polymer-amino carbon nanotube nanocomposites for surface acoustic wave CO2 detection. Rom. J. Inf. Sci. Technol..

[9] Wang R., Zhang M., Guan Y., Chen M., Zhang Y. (2019). A CO2-responsive hydrogel film for optical sensing of dissolved CO2. Soft Matter.

[10] Nazemi H., Joseph A., Park J., Emadi A. (2019). Advanced Micro- and Nano-Gas Sensor Technology: A Review. Sensors.

[11] Mo Y., Okawa Y., Tajima M., Nakai T., Yoshiike N., Natukawa K. (2001). Micro-machined gas sensor array based on metal film micro-heater. Sensors Actuators B Chem..

[12] Fang Q., Chetwynd D.G., Covington J.A., Toh C.S., Gardner J.W. (2002). Micro-gas-sensor with conducting polymers. Sensors Actuators B Chem..

[13] Aving (2013). TCC ELT, 19 New CO₂ Sensors which Last up to 10 Years. http://us.aving.net/news/view.php?articleId=718053.

[14] Fu X., Xu L. (2018). A Micro-Resonant Gas Sensor with Nanometer Clearance between the Pole Plates. Sensors.

[15] Hajjaj A.Z., Jaber N., Alcheikh N., Younis M.I. A Sensitive Resonant Gas Sensor Based on Multimode Excitation of a Buckled Beam. Proceedings of the 2019 20th International Conference on Solid-State Sensors, Actuators and Microsystems & Eurosensors XXXIII (TRANSDUCERS & EUROSENSORS XXXIII).

[16] Nguyen C.C., Ngo V.K.T., Le H.Q., Li W.L. (2019). Influences of relative humidity on the quality factors of MEMS cantilever resonators in gas rarefaction. Microsyst. Technol..

[17] Jaber N., Ilyas S., Shekhah O., Eddaoudi M., Younis M.I. (2018). Multimode MEMS Resonator for Simultaneous Sensing of Vapor Concentration and Temperature. IEEE Sensors J..

[18] Penza M., Aversa P., Cassano G., Wlodarski W., Kalantar-Zadeh K. (2007). Layered SAW gas sensor with single-walled carbon nanotube-based nanocomposite coating. Sensors Actuators B Chem..

[19] Fanget S., Hentz S., Puget P., Arcamone J., Matheron M., Colinet E., Andreucci P., Duraffourg L., Myers E., Roukes M.L. (2011). Gas sensors based on gravimetric detection—A review. Sensors Actuators B Chem..

[20] Smulko Janusz M. (2015). New approaches for improving selectivity and sensitivity of resistive gas sensors: A review. Sensor Rev..

[21] Sun L.-B., Kang Y.-H., Shi Y.-Q., Jiang Y., Liu X.-Q. (2015). Highly Selective Capture of the Greenhouse Gas CO2 in Polymers. ACS Sustain. Chem. Eng..

[22] Bouchaala A., Nayfeh A.H., Younis M.I. (2017). Analytical study of the frequency shifts of micro and nano clamped–clamped beam resonators due to an added mass. Meccanica.

[23] Liu F., Fu W., Chen S. (2019). Synthesis, characterization and CO2 adsorption performance of a thermosensitive solid amine adsorbent. J. CO2 Util..

[24] Ünveren E.E., Monkul B.Ö., Sarıoğlan Ş., Karademir N., Alper E. (2017). Solid amine sorbents for CO2 capture by chemical adsorption: A review. Petroleum.

[25] Irani M., Jacobson A.T., Gasem K.A.M., Fan M. (2018). Facilely synthesized porous polymer as support of poly(ethyleneimine) for effective CO2 capture. Energy.

[26] Sehaqui H., Gálvez M.E., Becatinni V., Cheng Ng Y., Steinfeld A., Zimmermann T., Tingaut P. (2015). Fast and Reversible Direct CO2 Capture from Air onto All-Polymer Nanofibrillated Cellulose—Polyethylenimine Foams. Environ. Sci. Technol..

[27] Ben Hamouda S., Roudesli S. (2008). Transport properties of PVA/PEI/PEG composite membranes: Sorption and permeation characterizations. Cent. Eur. J. Chem..

[28] Xian S., Wu Y., Wu J., Wang X., Xiao J. (2015). Enhanced Dynamic CO2 Adsorption Capacity and CO2/CH4 Selectivity on Polyethylenimine-Impregnated UiO-66. Ind. Eng. Chem. Res..

[29] Sun B., Xie G., Jiang Y., Li X. (2011). Comparative CO2-Sensing Characteristic Studies of PEI and PEI/Starch Thin Film Sensors. Energy Procedia.

[30] Bhanja P., Das S.K., Patra A.K., Bhaumik A. (2016). Functionalized graphene oxide as an efficient adsorbent for CO2 capture and support for heterogeneous catalysis. RSC Adv..

[31] Kim N., Xin G., Cho S.M., Pang C., Chae H. (2015). Microwave-reduced graphene oxide for efficient and stable hole extraction layers of polymer solar cells. Curr. Appl. Phys..

[32] Kato F., Noguchi H., Kodaka Y., Chiku N., Shibata H., Abe F., Ogi H. Multi-channel wireless quartz crystal microbalance biosensor fabricated with poly(dimethylsiloxane). Proceedings of the 2017 19th International Conference on Solid-State Sensors, Actuators and Microsystems (TRANSDUCERS).

[33] Branched Polyethylenimine by Sigma-Aldrich. https://www.sigmaaldrich.com/catalog/product/aldrich/408727?lang=en&region=CA.

[34] Lineal Polyethylenimine by Sigma-Aldrich. https://www.sigmaaldrich.com/catalog/product/aldrich/765090?lang=en&region=CA.

[35] Vieira R.B., Pastore H.O. (2014). Polyethylenimine-Magadiite Layered Silicate Sorbent for CO2 Capture. Environ. Sci. Technol..

[36] Yoon H.J., Jun D.H., Yang J.H., Zhou Z., Yang S.S., Cheng M.M.-C. (2011). Carbon dioxide gas sensor using a graphene sheet. Sensors Actuators B Chem..

[37] Basu S., Bhattacharyya P. (2012). Recent developments on graphene and graphene oxide based solid state gas sensors. Sensors Actuators B Chem..

[38] Chen W., Yan L., Bangal P.R. (2010). Preparation of graphene by the rapid and mild thermal reduction of graphene oxide induced by microwaves. Carbon.

[39] Pei S., Cheng H.-M. (2012). The reduction of graphene oxide. Carbon.

[40] Liu L., Zou G., Yang B., Luo X., Xu S. (2018). Amine-Functionalized Mesoporous Silica @ Reduced Graphene Sandwichlike Structure Composites for CO2 Adsorption. ACS Appl. Nano Mater..

[41] Zhang Y., Yu K., Xu R., Jiang D., Luo L., Zhu Z. (2005). Quartz crystal microbalance coated with carbon nanotube films used as humidity sensor. Sensors Actuators A Phys..

[42] Zhao R., Li X., Sun B., Li Y., Li Y., Yang R., Wang C. (2017). Branched polyethylenimine grafted electrospun polyacrylonitrile fiber membrane: A novel and effective adsorbent for Cr(vi) remediation in wastewater. J. Mater. Chem. A.

[43] Lott G.A., King M.D., Hill M.W., Scatena L.F. (2014). Effects of Relative Humidity on the Surface and Bulk Structures of Linear Polyethylenimine Thin Films. J. Phys. Chem. C.

[44] Wang X., Schwartz V., Clark J.C., Ma X., Overbury S.H., Xu X., Song C. (2009). Infrared Study of CO2 Sorption over “Molecular Basket” Sorbent Consisting of Polyethylenimine-Modified Mesoporous Molecular Sieve. J. Phys. Chem. C.

[45] Zhao Y., Ding H., Zhong Q. (2012). Preparation and characterization of aminated graphite oxide for CO2 capture. Appl. Surf. Sci..

[46] Song Y., Cao L., Yu J., Zhang S., Chen S., Jiang Y. (2017). Amino-functionalized graphene oxide blend with monoethanolamine for efficient carbon dioxide capture. J. Alloys Compd..

[47] Niu M., Yang H., Zhang X., Wang Y., Tang A. (2016). Amine-Impregnated Mesoporous Silica Nanotube as an Emerging Nanocomposite for CO2 Capture. ACS Appl. Mater. Interfaces.

[48] Cai H., Bao F., Gao J., Chen T., Wang S., Ma R. (2015). Preparation and characterization of novel carbon dioxide adsorbents based on polyethylenimine-modified Halloysite nanotubes. Environ. Technol..

[49] Zhang H., Goeppert A., Prakash G.K.S., Olah G. (2015). Applicability of linear polyethylenimine supported on nano-silica for the adsorption of CO2 from various sources including dry air. RSC Adv..

[50] Doan T.C.D., Baggerman J., Ramaneti R., Tong H.D., Marcelis A.T.M., van Rijn C.J.M. (2014). Carbon dioxide detection with polyethylenimine blended with polyelectrolytes. Sensors Actuators B Chem..

[51] Al-Marri M.J., Kuti Y.O., Khraisheh M., Kumar A., Khader M.M. (2017). Kinetics of CO2 Adsorption/Desorption of Polyethyleneimine-Mesoporous Silica. Chem. Eng. Technol..

[52] Aghehrochaboki R., Aghdoud Chaboki Y., Maleknia S.A., Irani V. (2019). Polyethyleneimine functionalized graphene oxide/methyldiethanolamine nanofluid: Preparation, characterization, and investigation of CO2 absorption. J. Environ. Chem. Eng..

[53] Andreoli E., Cullum L., Barron A.R. (2015). Carbon Dioxide Absorption by Polyethylenimine-Functionalized Nanocarbons: A Kinetic Study. Ind. Eng. Chem. Res..

[54] Muraoka S., Kiyohara Y., Oue H., Higashimoto S. (2014). A CO2 Sensor Using a Quartz Crystal Microbalance Coated with a Sensitive Membrane. Electron. Commun. Japan.

[55] Gomes M.T., Duarte A.C., Oliveira J.P. (1995). Detection of CO2 using a qaurtz crystal microbalance. Sensors Actuators B Chem..

[56] Ong K.G., Grimes C.A. (2001). A Carbon Nanotube-based Sensor for CO2 Monitoring. Sensors.

[57] Andò B., Baglio S., Di Pasquale G., Pollicino A., D’Agata S., Gugliuzzo C., Lombardo C., Re G. (2015). An Inkjet Printed CO2 Gas Sensor. Procedia Eng..

[58] Boudaden J., Klumpp A., Endres H.-E., Eisele I. (2019). Towards Low Cost and Low Temperature Capacitive CO2 Sensors Based on Amine Functionalized Silica Nanoparticles. Nanomaterials.

[59] Ma W., Wang R., Rong Q., Shao Z., Zhang W., Guo T., Wang J., Qiao X. (2017). CO2 Gas Sensing Using Optical Fiber Fabry–Perot Interferometer Based on Polyethyleneimine/Poly(Vinyl Alcohol) Coating. IEEE Photonics J..

[60] Clifford J., Mulrooney J., Dooly G., Fitzpatrick C., Lewis E., Merlone-Borla E., Flavia G. On board measurement of carbon dioxide exhaust car emissions using a mid-infrared optical based fibre. Proceedings of the SENSORS, 2008 IEEE.

[61] Sutela C., Collings N., Hands T. (1999). Fast Response CO2 Sensor for Automotive Exhaust Gas Analysis.

[62] Singh O.P., Howe T.A., Malarvili M.B. (2018). Real-time human respiration carbon dioxide measurement device for cardiorespiratory assessment. J. Breath Res..

